# 19-Ferrocenyl-18-oxa-8,16-diaza­penta­cyclo­[8.6.3.0^1,10^.0^2,7^.0^12,16^]nona­deca-2(7),3,5-triene-9,17-dione

**DOI:** 10.1107/S1600536810045459

**Published:** 2010-11-10

**Authors:** B. Gunasekaran, S. Kathiravan, R. Raghunathan, V. Manivannan

**Affiliations:** a Department of Physics, AMET University, Kanathur, Chennai 603 112, India; b Department of Organic Chemistry, University of Madras, Guindy campus, Chennai 600 025, India; c Department of Research and Development, PRIST University, Vallam, Thanjavur 613 403, Tamil Nadu, India

## Abstract

In the title compound, [Fe(C_5_H_5_)(C_21_H_19_N_2_O_3_)], both pyrrol­idine rings of the pyrrolizine substructure show an envelope conformation. In the ferrocenyl moiety, the unsubstituted cyclo­penta­dienyl ring is disordered over two orientations with site occupancies of 0.64 (2) and 0.36 (2). In the pyrrolizine ring, one C atom is disordered over two positions, with site occupancies of 0.71 (1) and 0.29 (1). Intra­molecular C—H⋯O inter­actions occur. The crystal packing is established through weak inter­molecular C—H⋯O and N—H⋯O inter­actions.

## Related literature

For the biological activity of ferrocene derivatives, see: Fouda *et al.* (2007 ▶); Jaouen *et al.* (2004 ▶); Biot *et al.* (2004 ▶). For related structures, see: Kamala *et al.* (2009 ▶); Gunasekaran *et al.* (2009 ▶);
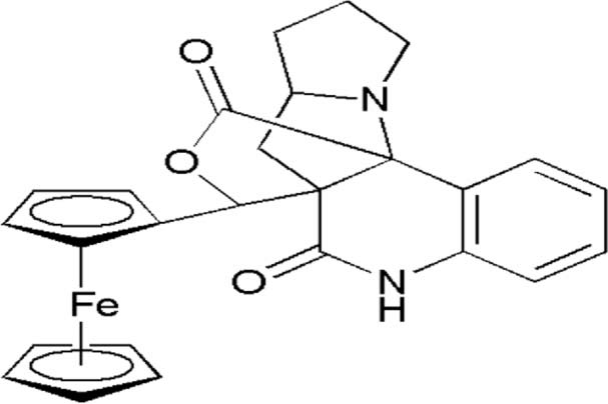


## Experimental

### 

#### Crystal data

[Fe(C_5_H_5_)(C_21_H_19_N_2_O_3_)]
*M* *_r_* = 468.32
Monoclinic, 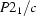

*a* = 9.6486 (7) Å
*b* = 18.8900 (13) Å
*c* = 11.8207 (9) Å
β = 93.248 (5)°
*V* = 2151.0 (3) Å^3^
*Z* = 4
Mo *K*α radiation
μ = 0.73 mm^−1^
*T* = 295 K
0.20 × 0.20 × 0.20 mm

#### Data collection

Bruker Kappa APEXII CCD diffractometer
Absorption correction: multi-scan (*SADABS*; Sheldrick, 1996 ▶) *T*_min_ = 0.864, *T*_max_ = 0.867
20500 measured reflections
5308 independent reflections
3322 reflections with *I* > 2σ(*I*)
*R*_int_ = 0.042

#### Refinement

*R*[*F* ^2^ > 2σ(*F* ^2^)] = 0.044
*wR*(*F* ^2^) = 0.123
*S* = 1.01
5308 reflections
344 parameters
5 restraints
H-atom parameters constrained
Δρ_max_ = 0.46 e Å^−3^
Δρ_min_ = −0.25 e Å^−3^

### 

Data collection: *APEX2* (Bruker, 2004 ▶); cell refinement: *SAINT* (Bruker, 2004 ▶); data reduction: *SAINT*; program(s) used to solve structure: *SHELXS97* (Sheldrick, 2008 ▶); program(s) used to refine structure: *SHELXL97* (Sheldrick, 2008 ▶); molecular graphics: *PLATON* (Spek, 2009 ▶); software used to prepare material for publication: *SHELXL97*.

## Supplementary Material

Crystal structure: contains datablocks global, I. DOI: 10.1107/S1600536810045459/im2240sup1.cif

Structure factors: contains datablocks I. DOI: 10.1107/S1600536810045459/im2240Isup2.hkl

Additional supplementary materials:  crystallographic information; 3D view; checkCIF report

## Figures and Tables

**Table 1:** Hydrogen-bond geometry (Å, °)

*D*—H⋯*A*	*D*—H	H⋯*A*	*D*⋯*A*	*D*—H⋯*A*
C10—H10*B*⋯O3	0.97	2.47	2.877 (3)	105
C9—H9⋯O1	0.93	2.58	3.128 (3)	118
C10*A*—H10*C*⋯O1^i^	0.98	2.56	3.371 (3)	140
N5—H5*A*⋯O3^ii^	0.86	1.99	2.840 (3)	168

Symmetry codes: (i) 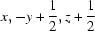; (ii) 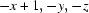.

**Fig. 1.**
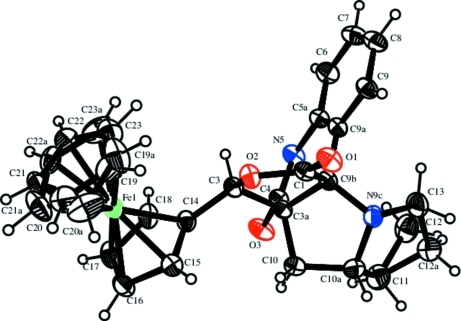
Molecular structure of (I), with atom labels and 30% probability displacement ellipsoids for non-H atoms.

**Fig. 2.**
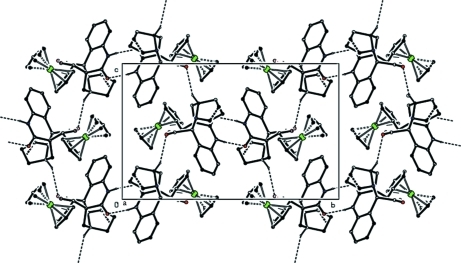
Crystal structure of (I), viewed down the a axis. Hydrogen bonds are shown as dashed lines.

## supplementary crystallographic information

### Comment

Metallocenes exhibit antibacterial (Fouda *et al.*, 2007), antitumor (Jaouen *et al.*, 2004), antimalarial and antifungal (Biot *et al.*, 2004) activities. The geometric parameters of the title compound (Fig. 1) agree well with reported similar structures (Kamala *et al.*, 2009; Gunasekaran *et al.*, 2009). N9c atom adopts *sp^3^* hybridization [sum of bond angles around N9c is 332.69 (2) °].

In the ferrocenyl moiety, one cyclopentadienyl (Cp) ring is disordered over two orientations [site occupancies of 0.64 (2) and 0.36 (2), respectively]. In the pyrrolizine ring one C atom is disordered over two positions [site occupancies of C12 and C12A are 0.71 (1) and 0.29 (1), respectively]. Crystal packing is established through weak intermolecular C—H···O and N—H···O interactions.

### Experimental

A mixture of methyl-2-hydroxy ferrocenyl methyl acrylate (1 mmol), isatin (1 mmol) and proline (1 mmol) was refluxed in methanol for 12 h until completion of the reaction was evidenced by TLC analysis. After completion of the reaction the solvent was evaporated under reduced pressure. The crude mixture was purified by column chromatography using ethyl acetate and hexane as eluent (10: 90). The product was dissolved in a methanol/chloroform mixture and heated for two minutes. The resulting solution was subjected to crystallization by slow evaporation of the solvents for 48 hours resulting in the formation of single crystals (yield 65%).

### Refinement

Site occupancy factors of disordered C atoms in the Cp ring were refined as 0.64 (C19-C23) and 0.36 (C19A-C23A). Disorder in the pyrrolizine system was refined to sof's of C12 = 0.71 (1) and C12A = 0.29 (1). Carbon-carbon bond distances in the disordered Cp ligand (C19A-C23A) were restrained to 1.450 (1) Å. H atoms were positioned geometrically and refined using riding model with C—H = 0.93 Å and *U*_iso_(H) = 1.2 *U_eq_*(C) for aromatic C—H groups, C—H = 0.98 Å and *U*_iso_(H) = 1.2 *U_eq_*(C) for methine groups, C—H = 0.97 Å and *U*_iso_(H) = 1.2 *U_eq_*(C) for CH_2_ and N—H = 0.86 Å and *U*_iso_(H) = 1.2 *U_eq_*(C) for the NH function of the quinoline moiety.

### Crystal data

**Table:**

[Fe(C_5_H_5_)(C_21_H_19_N_2_O_3_)]	*F*(000) = 976
*M**_r_* = 468.32	*D*_x_ = 1.446 Mg m^−^^3^
Monoclinic, *P*2_1_/*c*	Mo *K*α radiation, λ = 0.71073 Å
Hall symbol: -P 2ybc	Cell parameters from 4218 reflections
*a* = 9.6486 (7) Å	θ = 2.4–22.9°
*b* = 18.8900 (13) Å	µ = 0.73 mm^−^^1^
*c* = 11.8207 (9) Å	*T* = 295 K
β = 93.248 (5)°	Block, colourless
*V* = 2151.0 (3) Å^3^	0.20 × 0.20 × 0.20 mm
*Z* = 4	

### Data collection

**Table:**

Bruker Kappa APEXII CCD diffractometer	5308 independent reflections
Radiation source: fine-focus sealed tube	3322 reflections with *I* > 2σ(*I*)
graphite	*R*_int_ = 0.042
Detector resolution: 0 pixels mm^-1^	θ_max_ = 28.3°, θ_min_ = 2.0°
ω and φ scans	*h* = −12→12
Absorption correction: multi-scan (*SADABS*; Sheldrick, 1996)	*k* = −25→24
*T*_min_ = 0.864, *T*_max_ = 0.867	*l* = −15→15
20500 measured reflections	

### Refinement

**Table:**

Refinement on *F*^2^	Primary atom site location: structure-invariant direct methods
Least-squares matrix: full	Secondary atom site location: difference Fourier map
*R*[*F*^2^ > 2σ(*F*^2^)] = 0.044	Hydrogen site location: inferred from neighbouring sites
*wR*(*F*^2^) = 0.123	H-atom parameters constrained
*S* = 1.01	*w* = 1/[σ^2^(*F*_o_^2^) + (0.0546*P*)^2^ + 0.6413*P*] where *P* = (*F*_o_^2^ + 2*F*_c_^2^)/3
5308 reflections	(Δ/σ)_max_ < 0.001
344 parameters	Δρ_max_ = 0.46 e Å^−^^3^
5 restraints	Δρ_min_ = −0.24 e Å^−^^3^

### Fractional atomic coordinates and isotropic or equivalent isotropic displacement parameters (Å^2^)

**Table:**

	*x*	*y*	*z*	*U*_iso_*/*U*_eq_	Occ. (<1)
C14	0.4486 (2)	0.25037 (13)	0.0771 (2)	0.0425 (6)	
C15	0.4509 (3)	0.29893 (15)	0.1679 (2)	0.0548 (7)	
H15	0.5281	0.3232	0.1984	0.066*	
C16	0.3125 (3)	0.30383 (17)	0.2043 (3)	0.0636 (8)	
H16	0.2841	0.3322	0.2630	0.076*	
C17	0.2269 (3)	0.25912 (16)	0.1374 (3)	0.0633 (8)	
H17	0.1322	0.2524	0.1437	0.076*	
C18	0.3100 (3)	0.22591 (14)	0.0585 (3)	0.0517 (7)	
H18	0.2791	0.1935	0.0035	0.062*	
C3	0.5667 (2)	0.22556 (12)	0.0118 (2)	0.0395 (5)	
H3	0.5301	0.2093	−0.0627	0.047*	
C3A	0.6577 (2)	0.16611 (12)	0.0667 (2)	0.0364 (5)	
C9B	0.7977 (2)	0.17698 (12)	0.0127 (2)	0.0365 (5)	
C1	0.7951 (3)	0.25757 (12)	−0.0064 (2)	0.0396 (5)	
C10	0.6978 (3)	0.17695 (13)	0.1924 (2)	0.0437 (6)	
H10A	0.6952	0.2268	0.2122	0.052*	
H10B	0.6355	0.1512	0.2392	0.052*	
C10A	0.8450 (3)	0.14799 (14)	0.2076 (2)	0.0489 (6)	
H10C	0.8968	0.1752	0.2665	0.059*	
C11	0.8590 (4)	0.06879 (16)	0.2343 (3)	0.0718 (9)	
H11A	0.7689	0.0479	0.2451	0.086*	
H11B	0.9183	0.0613	0.3022	0.086*	
C12	0.9244 (7)	0.0365 (3)	0.1300 (5)	0.075 (2)	0.708 (12)
H12A	0.9907	−0.0002	0.1524	0.089*	0.708 (12)
H12B	0.8538	0.0166	0.0777	0.089*	0.708 (12)
C12A	0.9983 (15)	0.0539 (7)	0.1800 (12)	0.065 (4)*	0.292 (12)
H12C	1.0761	0.0679	0.2307	0.078*	0.292 (12)
H12D	1.0071	0.0041	0.1621	0.078*	0.292 (12)
C13	0.9927 (3)	0.09571 (18)	0.0797 (3)	0.0738 (10)	
H13A	1.0005	0.0876	−0.0007	0.089*	
H13B	1.0852	0.1018	0.1149	0.089*	
C9A	0.8044 (2)	0.14275 (12)	−0.1028 (2)	0.0382 (5)	
C9	0.9033 (3)	0.16238 (13)	−0.1781 (2)	0.0455 (6)	
H9	0.9671	0.1976	−0.1576	0.055*	
C8	0.9081 (3)	0.13042 (15)	−0.2823 (2)	0.0558 (7)	
H8	0.9746	0.1440	−0.3319	0.067*	
C7	0.8138 (3)	0.07823 (15)	−0.3128 (2)	0.0599 (8)	
H7	0.8159	0.0572	−0.3838	0.072*	
C6	0.7164 (3)	0.05681 (14)	−0.2394 (2)	0.0545 (7)	
H6	0.6537	0.0211	−0.2603	0.065*	
C5A	0.7126 (3)	0.08898 (12)	−0.1334 (2)	0.0412 (6)	
C4	0.5898 (2)	0.09531 (13)	0.0413 (2)	0.0414 (6)	
N9C	0.9090 (2)	0.15864 (11)	0.09766 (18)	0.0469 (5)	
N5	0.6147 (2)	0.06523 (11)	−0.05809 (18)	0.0474 (5)	
H5A	0.5670	0.0283	−0.0775	0.057*	
O2	0.66395 (17)	0.28264 (8)	−0.00449 (15)	0.0450 (4)	
O1	0.89048 (18)	0.29575 (9)	−0.02222 (15)	0.0506 (5)	
O3	0.5114 (2)	0.06791 (9)	0.10763 (16)	0.0592 (5)	
Fe1	0.31975 (4)	0.332977 (19)	0.03953 (3)	0.04542 (14)	
C19	0.4021 (12)	0.4127 (9)	−0.0405 (13)	0.074 (5)	0.64 (2)
H19	0.4950	0.4248	−0.0274	0.089*	0.64 (2)
C20	0.2958 (16)	0.4379 (6)	0.0168 (10)	0.071 (3)	0.64 (2)
H20	0.3017	0.4725	0.0728	0.086*	0.64 (2)
C21	0.1752 (11)	0.4029 (7)	−0.0230 (10)	0.059 (3)	0.64 (2)
H21	0.0871	0.4077	0.0042	0.071*	0.64 (2)
C22	0.2133 (15)	0.3587 (7)	−0.1127 (11)	0.062 (3)	0.64 (2)
H22	0.1561	0.3294	−0.1583	0.074*	0.64 (2)
C23	0.3532 (15)	0.3686 (8)	−0.1174 (12)	0.069 (5)	0.64 (2)
H23	0.4072	0.3464	−0.1695	0.083*	0.64 (2)
C21A	0.208 (2)	0.4262 (11)	0.025 (3)	0.082 (7)	0.36 (2)
H21A	0.1398	0.4446	0.0692	0.099*	0.36 (2)
C22A	0.1881 (14)	0.3788 (11)	−0.071 (2)	0.057 (6)	0.36 (2)
H22A	0.1027	0.3598	−0.0959	0.068*	0.36 (2)
C20A	0.356 (3)	0.4385 (14)	0.037 (2)	0.085 (7)	0.36 (2)
H20A	0.4040	0.4608	0.0970	0.103*	0.36 (2)
C19A	0.416 (2)	0.4096 (16)	−0.063 (3)	0.082 (10)	0.36 (2)
H19A	0.5055	0.4189	−0.0849	0.099*	0.36 (2)
C23A	0.317 (3)	0.3643 (17)	−0.125 (2)	0.082 (10)	0.36 (2)
H23A	0.3318	0.3334	−0.1841	0.098*	0.36 (2)

### Atomic displacement parameters (Å^2^)

**Table:**

	*U*^11^	*U*^22^	*U*^33^	*U*^12^	*U*^13^	*U*^23^
C14	0.0374 (13)	0.0417 (14)	0.0488 (15)	−0.0002 (10)	0.0060 (11)	0.0036 (11)
C15	0.0537 (16)	0.0603 (18)	0.0507 (17)	0.0089 (14)	0.0039 (13)	−0.0031 (14)
C16	0.068 (2)	0.071 (2)	0.0535 (18)	0.0225 (17)	0.0214 (15)	0.0048 (15)
C17	0.0494 (16)	0.0603 (18)	0.083 (2)	0.0066 (14)	0.0256 (16)	0.0236 (16)
C18	0.0410 (14)	0.0417 (14)	0.073 (2)	−0.0031 (11)	0.0070 (13)	0.0075 (13)
C3	0.0372 (12)	0.0375 (13)	0.0445 (14)	−0.0048 (10)	0.0072 (10)	−0.0004 (10)
C3A	0.0370 (12)	0.0345 (12)	0.0381 (13)	−0.0042 (10)	0.0074 (10)	−0.0013 (10)
C9B	0.0350 (12)	0.0332 (12)	0.0419 (14)	−0.0050 (9)	0.0086 (10)	0.0004 (10)
C1	0.0423 (13)	0.0396 (13)	0.0379 (13)	−0.0048 (10)	0.0100 (10)	−0.0018 (10)
C10	0.0490 (14)	0.0437 (14)	0.0390 (14)	−0.0039 (11)	0.0076 (11)	−0.0009 (11)
C10A	0.0528 (15)	0.0535 (16)	0.0401 (15)	0.0008 (12)	0.0002 (12)	−0.0028 (12)
C11	0.074 (2)	0.070 (2)	0.071 (2)	0.0129 (17)	0.0075 (17)	0.0261 (17)
C12	0.075 (4)	0.053 (3)	0.095 (4)	0.015 (2)	−0.005 (3)	−0.005 (3)
C13	0.071 (2)	0.082 (2)	0.071 (2)	0.0344 (18)	0.0142 (17)	0.0149 (18)
C9A	0.0403 (12)	0.0330 (12)	0.0421 (14)	−0.0007 (10)	0.0087 (10)	0.0004 (10)
C9	0.0498 (14)	0.0385 (13)	0.0499 (15)	−0.0008 (11)	0.0171 (12)	0.0034 (11)
C8	0.0698 (19)	0.0482 (16)	0.0519 (17)	0.0052 (14)	0.0269 (14)	0.0054 (13)
C7	0.080 (2)	0.0594 (18)	0.0417 (16)	0.0027 (16)	0.0164 (14)	−0.0074 (13)
C6	0.0620 (17)	0.0470 (15)	0.0551 (17)	−0.0063 (13)	0.0084 (14)	−0.0101 (13)
C5A	0.0460 (13)	0.0367 (13)	0.0414 (14)	−0.0017 (11)	0.0081 (11)	−0.0006 (10)
C4	0.0411 (13)	0.0366 (12)	0.0471 (15)	−0.0057 (10)	0.0082 (11)	−0.0017 (11)
N9C	0.0391 (11)	0.0504 (13)	0.0514 (13)	0.0026 (9)	0.0038 (10)	0.0067 (10)
N5	0.0500 (12)	0.0400 (12)	0.0532 (13)	−0.0152 (10)	0.0131 (10)	−0.0084 (10)
O2	0.0419 (9)	0.0347 (9)	0.0595 (11)	−0.0027 (7)	0.0114 (8)	0.0052 (8)
O1	0.0513 (11)	0.0442 (10)	0.0579 (12)	−0.0156 (8)	0.0165 (9)	−0.0021 (8)
O3	0.0684 (12)	0.0494 (11)	0.0628 (13)	−0.0222 (9)	0.0303 (10)	−0.0050 (9)
Fe1	0.0401 (2)	0.0419 (2)	0.0552 (3)	0.00089 (16)	0.01126 (16)	0.00516 (17)
C19	0.059 (6)	0.080 (9)	0.082 (8)	−0.029 (6)	−0.004 (7)	0.033 (6)
C20	0.110 (10)	0.038 (4)	0.066 (6)	0.015 (6)	0.002 (6)	−0.003 (3)
C21	0.064 (4)	0.056 (8)	0.060 (8)	0.027 (5)	0.027 (5)	0.015 (5)
C22	0.066 (6)	0.064 (6)	0.055 (6)	0.010 (4)	0.003 (4)	0.005 (4)
C23	0.072 (5)	0.064 (9)	0.075 (9)	0.010 (6)	0.033 (6)	0.029 (7)
C21A	0.103 (16)	0.052 (10)	0.098 (16)	0.029 (10)	0.059 (13)	0.014 (9)
C22A	0.035 (8)	0.057 (16)	0.08 (2)	−0.002 (9)	−0.002 (10)	0.019 (11)
C20A	0.119 (17)	0.059 (8)	0.074 (12)	−0.028 (11)	−0.022 (12)	0.014 (8)
C19A	0.068 (10)	0.055 (12)	0.13 (2)	0.003 (8)	0.063 (13)	0.043 (13)
C23A	0.105 (19)	0.072 (12)	0.064 (13)	−0.027 (14)	−0.030 (14)	−0.002 (9)

### Geometric parameters (Å, °)

**Table:**

C14—C15	1.411 (4)	C13—H13A	0.9700
C14—C18	1.421 (3)	C13—H13B	0.9700
C14—C3	1.488 (3)	C9A—C5A	1.382 (3)
C14—Fe1	2.029 (2)	C9A—C9	1.391 (3)
C15—C16	1.429 (4)	C9—C8	1.375 (4)
C15—Fe1	2.025 (3)	C9—H9	0.9300
C15—H15	0.9300	C8—C7	1.376 (4)
C16—C17	1.395 (4)	C8—H8	0.9300
C16—Fe1	2.029 (3)	C7—C6	1.375 (4)
C16—H16	0.9300	C7—H7	0.9300
C17—C18	1.411 (4)	C6—C5A	1.395 (3)
C17—Fe1	2.050 (3)	C6—H6	0.9300
C17—H17	0.9300	C5A—N5	1.407 (3)
C18—Fe1	2.038 (3)	C4—O3	1.233 (3)
C18—H18	0.9300	C4—N5	1.339 (3)
C3—O2	1.449 (3)	N5—H5A	0.8600
C3—C3A	1.546 (3)	Fe1—C19	1.970 (16)
C3—H3	0.9800	Fe1—C22A	1.973 (15)
C3A—C4	1.512 (3)	Fe1—C20	2.012 (11)
C3A—C10	1.528 (3)	Fe1—C23	2.016 (15)
C3A—C9B	1.540 (3)	Fe1—C20A	2.02 (3)
C9B—N9C	1.469 (3)	Fe1—C23A	2.03 (3)
C9B—C9A	1.516 (3)	Fe1—C21	2.030 (10)
C9B—C1	1.539 (3)	C19—C23	1.301 (14)
C1—O1	1.193 (3)	C19—C20	1.348 (14)
C1—O2	1.352 (3)	C19—H19	0.9300
C10—C10A	1.523 (4)	C20—C21	1.397 (15)
C10—H10A	0.9700	C20—H20	0.9300
C10—H10B	0.9700	C21—C22	1.415 (11)
C10A—N9C	1.484 (3)	C21—H21	0.9300
C10A—C11	1.533 (4)	C22—C23	1.367 (15)
C10A—H10C	0.9800	C22—H22	0.9300
C11—C12	1.542 (6)	C23—H23	0.9300
C11—C12A	1.547 (12)	C21A—C22A	1.4499 (10)
C11—H11A	0.9700	C21A—C20A	1.4499 (10)
C11—H11B	0.9700	C21A—H21A	0.9300
C12—C13	1.443 (6)	C22A—C23A	1.4499 (10)
C12—H12A	0.9700	C22A—H22A	0.9300
C12—H12B	0.9700	C20A—C19A	1.4499 (10)
C12A—C13	1.423 (12)	C20A—H20A	0.9300
C12A—H12C	0.9700	C19A—C23A	1.4500 (10)
C12A—H12D	0.9700	C19A—H19A	0.9300
C13—N9C	1.459 (4)	C23A—H23A	0.9300
			
C15—C14—C18	107.7 (2)	C19—Fe1—C23	38.1 (4)
C15—C14—C3	128.3 (2)	C22A—Fe1—C23	51.2 (7)
C18—C14—C3	124.0 (2)	C20—Fe1—C23	64.6 (5)
C15—C14—Fe1	69.49 (15)	C19—Fe1—C20A	33.3 (9)
C18—C14—Fe1	69.88 (14)	C22A—Fe1—C20A	70.3 (6)
C3—C14—Fe1	127.32 (17)	C20—Fe1—C20A	17.5 (5)
C14—C15—C16	107.2 (3)	C23—Fe1—C20A	67.5 (8)
C14—C15—Fe1	69.78 (15)	C19—Fe1—C15	110.5 (4)
C16—C15—Fe1	69.52 (17)	C22A—Fe1—C15	171.4 (9)
C14—C15—H15	126.4	C20—Fe1—C15	118.4 (4)
C16—C15—H15	126.4	C23—Fe1—C15	132.0 (4)
Fe1—C15—H15	125.9	C20A—Fe1—C15	103.0 (6)
C17—C16—C15	108.9 (3)	C19—Fe1—C14	115.8 (5)
C17—C16—Fe1	70.83 (17)	C22A—Fe1—C14	147.8 (9)
C15—C16—Fe1	69.22 (16)	C20—Fe1—C14	148.4 (4)
C17—C16—H16	125.6	C23—Fe1—C14	109.3 (4)
C15—C16—H16	125.6	C20A—Fe1—C14	131.0 (7)
Fe1—C16—H16	126.0	C15—Fe1—C14	40.74 (10)
C16—C17—C18	107.7 (3)	C19—Fe1—C23A	45.7 (9)
C16—C17—Fe1	69.18 (17)	C22A—Fe1—C23A	42.5 (4)
C18—C17—Fe1	69.32 (15)	C20—Fe1—C23A	65.8 (9)
C16—C17—H17	126.2	C23—Fe1—C23A	10.4 (8)
C18—C17—H17	126.2	C20A—Fe1—C23A	71.9 (9)
Fe1—C17—H17	126.9	C15—Fe1—C23A	141.7 (7)
C17—C18—C14	108.5 (3)	C14—Fe1—C23A	114.2 (8)
C17—C18—Fe1	70.30 (16)	C19—Fe1—C16	134.9 (4)
C14—C18—Fe1	69.22 (14)	C22A—Fe1—C16	134.5 (7)
C17—C18—H18	125.7	C20—Fe1—C16	112.7 (3)
C14—C18—H18	125.7	C23—Fe1—C16	171.7 (5)
Fe1—C18—H18	126.3	C20A—Fe1—C16	107.4 (7)
O2—C3—C14	110.80 (19)	C15—Fe1—C16	41.27 (11)
O2—C3—C3A	103.87 (18)	C14—Fe1—C16	68.58 (11)
C14—C3—C3A	116.4 (2)	C23A—Fe1—C16	177.0 (7)
O2—C3—H3	108.5	C19—Fe1—C21	67.4 (5)
C14—C3—H3	108.5	C22A—Fe1—C21	21.4 (6)
C3A—C3—H3	108.5	C20—Fe1—C21	40.4 (4)
C4—C3A—C10	113.23 (19)	C23—Fe1—C21	65.8 (5)
C4—C3A—C9B	114.61 (19)	C20A—Fe1—C21	57.9 (6)
C10—C3A—C9B	101.88 (19)	C15—Fe1—C21	150.1 (4)
C4—C3A—C3	109.24 (19)	C14—Fe1—C21	168.6 (4)
C10—C3A—C3	114.67 (19)	C23A—Fe1—C21	59.3 (7)
C9B—C3A—C3	102.71 (18)	C16—Fe1—C21	117.8 (3)
N9C—C9B—C9A	116.66 (19)	C19—Fe1—C18	146.5 (5)
N9C—C9B—C1	109.81 (19)	C22A—Fe1—C18	118.4 (6)
C9A—C9B—C1	106.91 (18)	C20—Fe1—C18	170.7 (4)
N9C—C9B—C3A	108.05 (18)	C23—Fe1—C18	116.3 (4)
C9A—C9B—C3A	113.53 (19)	C20A—Fe1—C18	171.2 (7)
C1—C9B—C3A	100.65 (18)	C15—Fe1—C18	68.49 (11)
O1—C1—O2	121.5 (2)	C14—Fe1—C18	40.90 (10)
O1—C1—C9B	127.9 (2)	C23A—Fe1—C18	113.3 (8)
O2—C1—C9B	110.60 (18)	C16—Fe1—C18	67.72 (13)
C10A—C10—C3A	104.41 (19)	C21—Fe1—C18	130.6 (4)
C10A—C10—H10A	110.9	C19—Fe1—C17	172.9 (5)
C3A—C10—H10A	110.9	C22A—Fe1—C17	112.8 (4)
C10A—C10—H10B	110.9	C20—Fe1—C17	134.1 (4)
C3A—C10—H10B	110.9	C23—Fe1—C17	147.6 (5)
H10A—C10—H10B	108.9	C20A—Fe1—C17	139.6 (8)
N9C—C10A—C10	106.3 (2)	C15—Fe1—C17	68.62 (12)
N9C—C10A—C11	106.0 (2)	C14—Fe1—C17	68.59 (10)
C10—C10A—C11	116.5 (2)	C23A—Fe1—C17	139.1 (8)
N9C—C10A—H10C	109.3	C16—Fe1—C17	39.99 (13)
C10—C10A—H10C	109.3	C21—Fe1—C17	109.6 (3)
C11—C10A—H10C	109.3	C18—Fe1—C17	40.38 (11)
C10A—C11—C12	104.9 (3)	C23—C19—C20	108.7 (9)
C10A—C11—C12A	99.2 (5)	C23—C19—Fe1	72.9 (9)
C10A—C11—H11A	110.8	C20—C19—Fe1	71.9 (8)
C12—C11—H11A	110.8	C23—C19—H19	125.7
C12A—C11—H11A	141.9	C20—C19—H19	125.7
C10A—C11—H11B	110.8	Fe1—C19—H19	121.3
C12—C11—H11B	110.8	C19—C20—C21	107.9 (7)
C12A—C11—H11B	80.3	C19—C20—Fe1	68.5 (8)
H11A—C11—H11B	108.8	C21—C20—Fe1	70.4 (6)
C13—C12—C11	103.9 (4)	C19—C20—H20	126.1
C13—C12—H12A	111.0	C21—C20—H20	126.1
C11—C12—H12A	111.0	Fe1—C20—H20	126.5
C13—C12—H12B	111.0	C20—C21—C22	106.6 (10)
C11—C12—H12B	111.0	C20—C21—Fe1	69.1 (6)
H12A—C12—H12B	109.0	C22—C21—Fe1	71.7 (8)
C13—C12A—C11	104.6 (8)	C20—C21—H21	126.7
C13—C12A—H12C	110.8	C22—C21—H21	126.7
C11—C12A—H12C	110.8	Fe1—C21—H21	124.1
C13—C12A—H12D	110.8	C23—C22—C21	104.3 (14)
C11—C12A—H12D	110.8	C23—C22—Fe1	68.0 (9)
H12C—C12A—H12D	108.9	C21—C22—Fe1	68.0 (8)
C12A—C13—N9C	109.0 (5)	C23—C22—H22	127.8
C12—C13—N9C	107.6 (3)	C21—C22—H22	127.8
C12A—C13—H13A	136.7	Fe1—C22—H22	127.6
C12—C13—H13A	110.2	C19—C23—C22	112.4 (12)
N9C—C13—H13A	110.2	C19—C23—Fe1	69.1 (9)
C12A—C13—H13B	73.6	C22—C23—Fe1	73.0 (8)
C12—C13—H13B	110.2	C19—C23—H23	123.8
N9C—C13—H13B	110.2	C22—C23—H23	123.8
H13A—C13—H13B	108.5	Fe1—C23—H23	125.7
C5A—C9A—C9	118.8 (2)	C22A—C21A—C20A	105.0 (15)
C5A—C9A—C9B	119.3 (2)	C22A—C21A—Fe1	65.5 (9)
C9—C9A—C9B	121.9 (2)	C20A—C21A—Fe1	67.6 (13)
C8—C9—C9A	121.0 (2)	C22A—C21A—H21A	127.5
C8—C9—H9	119.5	C20A—C21A—H21A	127.5
C9A—C9—H9	119.5	Fe1—C21A—H21A	130.8
C9—C8—C7	119.6 (3)	C21A—C22A—C23A	112.4 (14)
C9—C8—H8	120.2	C21A—C22A—Fe1	72.5 (10)
C7—C8—H8	120.2	C23A—C22A—Fe1	70.8 (15)
C6—C7—C8	120.8 (3)	C21A—C22A—H22A	123.8
C6—C7—H7	119.6	C23A—C22A—H22A	123.8
C8—C7—H7	119.6	Fe1—C22A—H22A	124.4
C7—C6—C5A	119.5 (3)	C21A—C20A—C19A	107.4 (17)
C7—C6—H6	120.2	C21A—C20A—Fe1	70.9 (12)
C5A—C6—H6	120.2	C19A—C20A—Fe1	73.9 (16)
C9A—C5A—C6	120.4 (2)	C21A—C20A—H20A	126.3
C9A—C5A—N5	120.8 (2)	C19A—C20A—H20A	126.3
C6—C5A—N5	118.9 (2)	Fe1—C20A—H20A	120.8
O3—C4—N5	121.8 (2)	C20A—C19A—C23A	110.3 (18)
O3—C4—C3A	121.3 (2)	C20A—C19A—Fe1	65.5 (15)
N5—C4—C3A	116.9 (2)	C23A—C19A—Fe1	65.7 (17)
C13—N9C—C9B	118.8 (2)	C20A—C19A—H19A	124.9
C13—N9C—C10A	106.1 (2)	C23A—C19A—H19A	124.9
C9B—N9C—C10A	107.79 (18)	Fe1—C19A—H19A	136.3
C4—N5—C5A	125.5 (2)	C22A—C23A—C19A	103.2 (15)
C4—N5—H5A	117.3	C22A—C23A—Fe1	66.7 (12)
C5A—N5—H5A	117.3	C19A—C23A—Fe1	73.7 (17)
C1—O2—C3	110.79 (17)	C22A—C23A—H23A	128.4
C19—Fe1—C22A	66.9 (5)	C19A—C23A—H23A	128.4
C19—Fe1—C20	39.5 (5)	Fe1—C23A—H23A	123.0
C22A—Fe1—C20	54.1 (5)		
			
C18—C14—C15—C16	0.0 (3)	C20A—Fe1—C19—C20	−27.8 (10)
C3—C14—C15—C16	−178.4 (2)	C15—Fe1—C19—C20	−110.1 (8)
Fe1—C14—C15—C16	59.68 (19)	C14—Fe1—C19—C20	−154.2 (7)
C18—C14—C15—Fe1	−59.70 (18)	C23A—Fe1—C19—C20	106.3 (12)
C3—C14—C15—Fe1	122.0 (3)	C16—Fe1—C19—C20	−69.5 (9)
C14—C15—C16—C17	0.1 (3)	C21—Fe1—C19—C20	37.8 (6)
Fe1—C15—C16—C17	59.9 (2)	C18—Fe1—C19—C20	168.2 (7)
C14—C15—C16—Fe1	−59.85 (19)	C23—C19—C20—C21	4.3 (17)
C15—C16—C17—C18	−0.1 (3)	Fe1—C19—C20—C21	−59.7 (8)
Fe1—C16—C17—C18	58.81 (19)	C23—C19—C20—Fe1	64.0 (13)
C15—C16—C17—Fe1	−58.9 (2)	C22A—Fe1—C20—C19	−96.6 (8)
C16—C17—C18—C14	0.1 (3)	C23—Fe1—C20—C19	−37.5 (6)
Fe1—C17—C18—C14	58.84 (18)	C20A—Fe1—C20—C19	58 (3)
C16—C17—C18—Fe1	−58.7 (2)	C15—Fe1—C20—C19	88.4 (7)
C15—C14—C18—C17	−0.1 (3)	C14—Fe1—C20—C19	48.3 (10)
C3—C14—C18—C17	178.4 (2)	C23A—Fe1—C20—C19	−48.9 (9)
Fe1—C14—C18—C17	−59.51 (19)	C16—Fe1—C20—C19	134.0 (7)
C15—C14—C18—Fe1	59.45 (18)	C21—Fe1—C20—C19	−119.3 (8)
C3—C14—C18—Fe1	−122.1 (2)	C17—Fe1—C20—C19	175.3 (6)
C15—C14—C3—O2	−37.1 (4)	C19—Fe1—C20—C21	119.3 (8)
C18—C14—C3—O2	144.9 (2)	C22A—Fe1—C20—C21	22.7 (7)
Fe1—C14—C3—O2	55.3 (3)	C23—Fe1—C20—C21	81.8 (6)
C15—C14—C3—C3A	81.3 (3)	C20A—Fe1—C20—C21	178 (3)
C18—C14—C3—C3A	−96.8 (3)	C15—Fe1—C20—C21	−152.3 (5)
Fe1—C14—C3—C3A	173.62 (16)	C14—Fe1—C20—C21	167.6 (6)
O2—C3—C3A—C4	−154.81 (18)	C23A—Fe1—C20—C21	70.4 (8)
C14—C3—C3A—C4	83.1 (3)	C16—Fe1—C20—C21	−106.7 (5)
O2—C3—C3A—C10	76.9 (2)	C17—Fe1—C20—C21	−65.4 (6)
C14—C3—C3A—C10	−45.2 (3)	C19—C20—C21—C22	−3.9 (16)
O2—C3—C3A—C9B	−32.7 (2)	Fe1—C20—C21—C22	−62.4 (10)
C14—C3—C3A—C9B	−154.8 (2)	C19—C20—C21—Fe1	58.5 (10)
C4—C3A—C9B—N9C	−96.0 (2)	C19—Fe1—C21—C20	−37.0 (5)
C10—C3A—C9B—N9C	26.7 (2)	C22A—Fe1—C21—C20	−121.2 (14)
C3—C3A—C9B—N9C	145.68 (18)	C23—Fe1—C21—C20	−78.6 (7)
C4—C3A—C9B—C9A	35.1 (3)	C20A—Fe1—C21—C20	−0.9 (12)
C10—C3A—C9B—C9A	157.72 (19)	C15—Fe1—C21—C20	55.2 (8)
C3—C3A—C9B—C9A	−83.3 (2)	C14—Fe1—C21—C20	−145.3 (14)
C4—C3A—C9B—C1	148.9 (2)	C23A—Fe1—C21—C20	−87.8 (10)
C10—C3A—C9B—C1	−88.4 (2)	C16—Fe1—C21—C20	93.1 (6)
C3—C3A—C9B—C1	30.6 (2)	C18—Fe1—C21—C20	176.6 (5)
N9C—C9B—C1—O1	47.7 (3)	C17—Fe1—C21—C20	136.1 (6)
C9A—C9B—C1—O1	−79.7 (3)	C19—Fe1—C21—C22	79.6 (9)
C3A—C9B—C1—O1	161.5 (2)	C22A—Fe1—C21—C22	−4.6 (13)
N9C—C9B—C1—O2	−133.0 (2)	C20—Fe1—C21—C22	116.6 (10)
C9A—C9B—C1—O2	99.6 (2)	C23—Fe1—C21—C22	38.0 (9)
C3A—C9B—C1—O2	−19.2 (2)	C20A—Fe1—C21—C22	115.7 (12)
C4—C3A—C10—C10A	89.9 (2)	C15—Fe1—C21—C22	171.7 (6)
C9B—C3A—C10—C10A	−33.7 (2)	C14—Fe1—C21—C22	−29 (2)
C3—C3A—C10—C10A	−143.8 (2)	C23A—Fe1—C21—C22	28.8 (10)
C3A—C10—C10A—N9C	29.6 (2)	C16—Fe1—C21—C22	−150.3 (8)
C3A—C10—C10A—C11	−88.2 (3)	C18—Fe1—C21—C22	−66.8 (9)
N9C—C10A—C11—C12	−3.7 (4)	C17—Fe1—C21—C22	−107.3 (8)
C10—C10A—C11—C12	114.2 (4)	C20—C21—C22—C23	2.0 (17)
N9C—C10A—C11—C12A	32.8 (7)	Fe1—C21—C22—C23	−58.7 (11)
C10—C10A—C11—C12A	150.8 (7)	C20—C21—C22—Fe1	60.7 (8)
C10A—C11—C12—C13	22.7 (5)	C19—Fe1—C22—C23	34.4 (9)
C12A—C11—C12—C13	−62.6 (8)	C22A—Fe1—C22—C23	122 (2)
C10A—C11—C12A—C13	−38.1 (10)	C20—Fe1—C22—C23	77.7 (9)
C12—C11—C12A—C13	64.5 (9)	C20A—Fe1—C22—C23	66.1 (11)
C11—C12A—C13—C12	−64.6 (9)	C15—Fe1—C22—C23	−42 (3)
C11—C12A—C13—N9C	30.2 (11)	C14—Fe1—C22—C23	−70.2 (11)
C11—C12—C13—C12A	64.6 (8)	C23A—Fe1—C22—C23	−4(2)
C11—C12—C13—N9C	−34.1 (5)	C16—Fe1—C22—C23	173.7 (6)
N9C—C9B—C9A—C5A	106.3 (2)	C21—Fe1—C22—C23	116.8 (14)
C1—C9B—C9A—C5A	−130.4 (2)	C18—Fe1—C22—C23	−110.3 (9)
C3A—C9B—C9A—C5A	−20.4 (3)	C17—Fe1—C22—C23	−153.0 (8)
N9C—C9B—C9A—C9	−71.7 (3)	C19—Fe1—C22—C21	−82.4 (9)
C1—C9B—C9A—C9	51.6 (3)	C22A—Fe1—C22—C21	5.2 (14)
C3A—C9B—C9A—C9	161.7 (2)	C20—Fe1—C22—C21	−39.1 (8)
C5A—C9A—C9—C8	1.7 (4)	C23—Fe1—C22—C21	−116.8 (14)
C9B—C9A—C9—C8	179.6 (2)	C20A—Fe1—C22—C21	−50.8 (10)
C9A—C9—C8—C7	0.0 (4)	C15—Fe1—C22—C21	−158.8 (15)
C9—C8—C7—C6	−1.2 (4)	C14—Fe1—C22—C21	172.9 (6)
C8—C7—C6—C5A	0.7 (4)	C23A—Fe1—C22—C21	−121 (2)
C9—C9A—C5A—C6	−2.1 (4)	C16—Fe1—C22—C21	56.9 (13)
C9B—C9A—C5A—C6	179.8 (2)	C18—Fe1—C22—C21	132.9 (8)
C9—C9A—C5A—N5	177.2 (2)	C17—Fe1—C22—C21	90.1 (9)
C9B—C9A—C5A—N5	−0.8 (3)	C20—C19—C23—C22	−3(2)
C7—C6—C5A—C9A	1.0 (4)	Fe1—C19—C23—C22	60.3 (13)
C7—C6—C5A—N5	−178.4 (2)	C20—C19—C23—Fe1	−63.4 (11)
C10—C3A—C4—O3	35.8 (3)	C21—C22—C23—C19	1(2)
C9B—C3A—C4—O3	152.1 (2)	Fe1—C22—C23—C19	−58.0 (13)
C3—C3A—C4—O3	−93.3 (3)	C21—C22—C23—Fe1	58.7 (10)
C10—C3A—C4—N5	−146.5 (2)	C22A—Fe1—C23—C19	102.2 (12)
C9B—C3A—C4—N5	−30.3 (3)	C20—Fe1—C23—C19	38.9 (8)
C3—C3A—C4—N5	84.3 (3)	C20A—Fe1—C23—C19	20.0 (10)
C12A—C13—N9C—C9B	−130.2 (8)	C15—Fe1—C23—C19	−67.4 (10)
C12—C13—N9C—C9B	−89.0 (4)	C14—Fe1—C23—C19	−107.4 (9)
C12A—C13—N9C—C10A	−8.7 (8)	C23A—Fe1—C23—C19	133 (7)
C12—C13—N9C—C10A	32.5 (4)	C21—Fe1—C23—C19	83.7 (9)
C9A—C9B—N9C—C13	−17.6 (3)	C18—Fe1—C23—C19	−151.4 (8)
C1—C9B—N9C—C13	−139.4 (2)	C17—Fe1—C23—C19	172.4 (8)
C3A—C9B—N9C—C13	111.7 (2)	C19—Fe1—C23—C22	−122.9 (13)
C9A—C9B—N9C—C10A	−138.3 (2)	C22A—Fe1—C23—C22	−20.7 (9)
C1—C9B—N9C—C10A	99.9 (2)	C20—Fe1—C23—C22	−84.0 (9)
C3A—C9B—N9C—C10A	−9.0 (2)	C20A—Fe1—C23—C22	−102.9 (10)
C10—C10A—N9C—C13	−141.1 (2)	C15—Fe1—C23—C22	169.8 (7)
C11—C10A—N9C—C13	−16.5 (3)	C14—Fe1—C23—C22	129.7 (8)
C10—C10A—N9C—C9B	−12.8 (2)	C23A—Fe1—C23—C22	10 (6)
C11—C10A—N9C—C9B	111.7 (2)	C21—Fe1—C23—C22	−39.2 (8)
O3—C4—N5—C5A	−173.3 (2)	C18—Fe1—C23—C22	85.8 (9)
C3A—C4—N5—C5A	9.0 (4)	C17—Fe1—C23—C22	49.5 (13)
C9A—C5A—N5—C4	7.5 (4)	C19—Fe1—C21A—C22A	85.7 (10)
C6—C5A—N5—C4	−173.1 (2)	C20—Fe1—C21A—C22A	107.9 (19)
O1—C1—O2—C3	177.8 (2)	C23—Fe1—C21A—C22A	47.2 (9)
C9B—C1—O2—C3	−1.6 (3)	C20A—Fe1—C21A—C22A	119.6 (15)
C14—C3—O2—C1	147.5 (2)	C15—Fe1—C21A—C22A	−179.8 (7)
C3A—C3—O2—C1	21.8 (2)	C14—Fe1—C21A—C22A	166 (3)
C14—C15—Fe1—C19	−106.0 (5)	C23A—Fe1—C21A—C22A	36.9 (9)
C16—C15—Fe1—C19	135.6 (5)	C16—Fe1—C21A—C22A	−141.3 (9)
C14—C15—Fe1—C20	−148.8 (4)	C21—Fe1—C21A—C22A	−13.2 (14)
C16—C15—Fe1—C20	92.8 (5)	C18—Fe1—C21A—C22A	−68.4 (14)
C14—C15—Fe1—C23	−68.6 (6)	C17—Fe1—C21A—C22A	−99.6 (9)
C16—C15—Fe1—C23	173.1 (6)	C19—Fe1—C21A—C20A	−34.0 (13)
C14—C15—Fe1—C20A	−139.9 (9)	C22A—Fe1—C21A—C20A	−119.6 (15)
C16—C15—Fe1—C20A	101.7 (9)	C20—Fe1—C21A—C20A	−11.7 (17)
C16—C15—Fe1—C14	−118.3 (2)	C23—Fe1—C21A—C20A	−72.5 (13)
C14—C15—Fe1—C23A	−62.8 (13)	C15—Fe1—C21A—C20A	60.6 (15)
C16—C15—Fe1—C23A	178.9 (13)	C14—Fe1—C21A—C20A	46 (4)
C14—C15—Fe1—C16	118.3 (2)	C23A—Fe1—C21A—C20A	−82.7 (12)
C14—C15—Fe1—C21	173.9 (6)	C16—Fe1—C21A—C20A	99.0 (14)
C16—C15—Fe1—C21	55.6 (7)	C21—Fe1—C21A—C20A	−133 (2)
C14—C15—Fe1—C18	38.06 (15)	C18—Fe1—C21A—C20A	171.9 (11)
C16—C15—Fe1—C18	−80.28 (19)	C17—Fe1—C21A—C20A	140.8 (13)
C14—C15—Fe1—C17	81.59 (17)	C20A—C21A—C22A—C23A	−4(3)
C16—C15—Fe1—C17	−36.75 (18)	Fe1—C21A—C22A—C23A	−59.9 (19)
C15—C14—Fe1—C19	92.1 (5)	C20A—C21A—C22A—Fe1	56.3 (15)
C18—C14—Fe1—C19	−149.0 (5)	C19—Fe1—C22A—C21A	−73.4 (10)
C3—C14—Fe1—C19	−31.1 (5)	C20—Fe1—C22A—C21A	−29.9 (10)
C15—C14—Fe1—C22A	179.2 (8)	C23—Fe1—C22A—C21A	−114.3 (12)
C18—C14—Fe1—C22A	−62.0 (8)	C20A—Fe1—C22A—C21A	−37.7 (9)
C3—C14—Fe1—C22A	56.0 (8)	C14—Fe1—C22A—C21A	−175.4 (8)
C15—C14—Fe1—C20	60.3 (6)	C23A—Fe1—C22A—C21A	−122.2 (16)
C18—C14—Fe1—C20	179.1 (6)	C16—Fe1—C22A—C21A	57.6 (11)
C3—C14—Fe1—C20	−62.9 (7)	C21—Fe1—C22A—C21A	13.3 (12)
C15—C14—Fe1—C23	132.9 (5)	C18—Fe1—C22A—C21A	143.5 (9)
C18—C14—Fe1—C23	−108.3 (5)	C17—Fe1—C22A—C21A	98.9 (10)
C3—C14—Fe1—C23	9.7 (5)	C19—Fe1—C22A—C23A	48.8 (13)
C15—C14—Fe1—C20A	56.3 (10)	C20—Fe1—C22A—C23A	92.3 (13)
C18—C14—Fe1—C20A	175.1 (10)	C23—Fe1—C22A—C23A	7.9 (15)
C3—C14—Fe1—C20A	−66.9 (10)	C20A—Fe1—C22A—C23A	84.4 (12)
C18—C14—Fe1—C15	118.8 (2)	C14—Fe1—C22A—C23A	−53.2 (16)
C3—C14—Fe1—C15	−123.2 (3)	C16—Fe1—C22A—C23A	179.8 (12)
C15—C14—Fe1—C23A	142.8 (8)	C21—Fe1—C22A—C23A	135.5 (19)
C18—C14—Fe1—C23A	−98.4 (8)	C18—Fe1—C22A—C23A	−94.3 (13)
C3—C14—Fe1—C23A	19.6 (9)	C17—Fe1—C22A—C23A	−138.9 (13)
C15—C14—Fe1—C16	−38.58 (18)	C22A—C21A—C20A—C19A	10 (3)
C18—C14—Fe1—C16	80.26 (19)	Fe1—C21A—C20A—C19A	65 (2)
C3—C14—Fe1—C16	−161.8 (3)	C22A—C21A—C20A—Fe1	−54.9 (13)
C15—C14—Fe1—C21	−164.6 (14)	C19—Fe1—C20A—C21A	115.8 (17)
C18—C14—Fe1—C21	−45.7 (14)	C22A—Fe1—C20A—C21A	38.1 (9)
C3—C14—Fe1—C21	72.3 (14)	C20—Fe1—C20A—C21A	17 (2)
C15—C14—Fe1—C18	−118.8 (2)	C23—Fe1—C20A—C21A	93.2 (13)
C3—C14—Fe1—C18	118.0 (3)	C15—Fe1—C20A—C21A	−136.4 (14)
C15—C14—Fe1—C17	−81.68 (18)	C14—Fe1—C20A—C21A	−170.2 (12)
C18—C14—Fe1—C17	37.16 (18)	C23A—Fe1—C20A—C21A	83.1 (11)
C3—C14—Fe1—C17	155.1 (3)	C16—Fe1—C20A—C21A	−93.8 (14)
C17—C16—Fe1—C19	172.7 (7)	C21—Fe1—C20A—C21A	18.6 (11)
C15—C16—Fe1—C19	−67.5 (7)	C17—Fe1—C20A—C21A	−64.2 (15)
C17—C16—Fe1—C22A	71.0 (10)	C19—Fe1—C20A—C19A	0.4 (17)
C15—C16—Fe1—C22A	−169.1 (9)	C22A—Fe1—C20A—C19A	−77.3 (13)
C17—C16—Fe1—C20	132.4 (5)	C20—Fe1—C20A—C19A	−99 (3)
C15—C16—Fe1—C20	−107.7 (5)	C23—Fe1—C20A—C19A	−22.3 (14)
C17—C16—Fe1—C20A	150.2 (8)	C15—Fe1—C20A—C19A	108.2 (14)
C15—C16—Fe1—C20A	−89.9 (8)	C14—Fe1—C20A—C19A	74.3 (16)
C17—C16—Fe1—C15	−119.9 (2)	C23A—Fe1—C20A—C19A	−32.4 (12)
C17—C16—Fe1—C14	−81.80 (18)	C16—Fe1—C20A—C19A	150.8 (14)
C15—C16—Fe1—C14	38.09 (16)	C21—Fe1—C20A—C19A	−96.9 (14)
C17—C16—Fe1—C21	87.8 (5)	C17—Fe1—C20A—C19A	−179.7 (11)
C15—C16—Fe1—C21	−152.3 (5)	C21A—C20A—C19A—C23A	−14 (3)
C17—C16—Fe1—C18	−37.58 (16)	Fe1—C20A—C19A—C23A	49 (2)
C15—C16—Fe1—C18	82.31 (18)	C21A—C20A—C19A—Fe1	−63.4 (17)
C15—C16—Fe1—C17	119.9 (2)	C19—Fe1—C19A—C20A	−2(7)
C17—C18—Fe1—C19	176.9 (7)	C22A—Fe1—C19A—C20A	86.0 (13)
C14—C18—Fe1—C19	57.1 (7)	C20—Fe1—C19A—C20A	24.4 (10)
C17—C18—Fe1—C22A	−92.5 (9)	C23—Fe1—C19A—C20A	139 (2)
C14—C18—Fe1—C22A	147.7 (9)	C15—Fe1—C19A—C20A	−84.8 (15)
C17—C18—Fe1—C23	−150.7 (5)	C14—Fe1—C19A—C20A	−128.7 (13)
C14—C18—Fe1—C23	89.5 (5)	C23A—Fe1—C19A—C20A	129 (2)
C17—C18—Fe1—C15	81.9 (2)	C16—Fe1—C19A—C20A	−47 (2)
C14—C18—Fe1—C15	−37.91 (15)	C21—Fe1—C19A—C20A	63.3 (12)
C17—C18—Fe1—C14	119.8 (3)	C18—Fe1—C19A—C20A	−166.5 (10)
C17—C18—Fe1—C23A	−139.7 (8)	C19—Fe1—C19A—C23A	−130 (9)
C14—C18—Fe1—C23A	100.5 (8)	C22A—Fe1—C19A—C23A	−42.6 (12)
C17—C18—Fe1—C16	37.23 (18)	C20—Fe1—C19A—C23A	−104.3 (17)
C14—C18—Fe1—C16	−82.55 (18)	C23—Fe1—C19A—C23A	10.2 (17)
C17—C18—Fe1—C21	−71.0 (5)	C20A—Fe1—C19A—C23A	−129 (2)
C14—C18—Fe1—C21	169.3 (4)	C15—Fe1—C19A—C23A	146.6 (14)
C14—C18—Fe1—C17	−119.8 (3)	C14—Fe1—C19A—C23A	102.6 (15)
C16—C17—Fe1—C22A	−133.0 (10)	C16—Fe1—C19A—C23A	−175.5 (12)
C18—C17—Fe1—C22A	107.6 (10)	C21—Fe1—C19A—C23A	−65.3 (13)
C16—C17—Fe1—C20	−71.5 (6)	C18—Fe1—C19A—C23A	64.9 (19)
C18—C17—Fe1—C20	169.1 (6)	C21A—C22A—C23A—C19A	−5(3)
C16—C17—Fe1—C23	174.4 (8)	Fe1—C22A—C23A—C19A	−65 (2)
C18—C17—Fe1—C23	54.9 (8)	C21A—C22A—C23A—Fe1	60.9 (15)
C16—C17—Fe1—C20A	−47.1 (10)	C20A—C19A—C23A—C22A	11 (4)
C18—C17—Fe1—C20A	−166.5 (10)	Fe1—C19A—C23A—C22A	60.5 (18)
C16—C17—Fe1—C15	37.89 (17)	C20A—C19A—C23A—Fe1	−49 (2)
C18—C17—Fe1—C15	−81.52 (18)	C19—Fe1—C23A—C22A	−104.7 (16)
C16—C17—Fe1—C14	81.79 (18)	C20—Fe1—C23A—C22A	−62.6 (11)
C18—C17—Fe1—C14	−37.62 (16)	C23—Fe1—C23A—C22A	−144 (7)
C16—C17—Fe1—C23A	−175.5 (12)	C20A—Fe1—C23A—C22A	−80.3 (11)
C18—C17—Fe1—C23A	65.0 (12)	C15—Fe1—C23A—C22A	−168.4 (11)
C18—C17—Fe1—C16	−119.4 (3)	C14—Fe1—C23A—C22A	152.1 (12)
C16—C17—Fe1—C21	−110.2 (5)	C21—Fe1—C23A—C22A	−17.3 (8)
C18—C17—Fe1—C21	130.4 (5)	C18—Fe1—C23A—C22A	107.3 (13)
C16—C17—Fe1—C18	119.4 (3)	C17—Fe1—C23A—C22A	67.5 (16)
C22A—Fe1—C19—C23	−55.9 (11)	C19—Fe1—C23A—C19A	7.9 (17)
C20—Fe1—C19—C23	−117.0 (11)	C22A—Fe1—C23A—C19A	112.6 (19)
C20A—Fe1—C19—C23	−144.8 (16)	C20—Fe1—C23A—C19A	50.0 (14)
C15—Fe1—C19—C23	133.0 (8)	C23—Fe1—C23A—C19A	−31 (6)
C14—Fe1—C19—C23	88.8 (9)	C20A—Fe1—C23A—C19A	32.4 (12)
C23A—Fe1—C19—C23	−10.7 (14)	C15—Fe1—C23A—C19A	−56 (2)
C16—Fe1—C19—C23	173.5 (7)	C14—Fe1—C23A—C19A	−95.2 (15)
C21—Fe1—C19—C23	−79.2 (8)	C21—Fe1—C23A—C19A	95.3 (15)
C18—Fe1—C19—C23	51.2 (12)	C18—Fe1—C23A—C19A	−140.1 (14)
C22A—Fe1—C19—C20	61.1 (8)	C17—Fe1—C23A—C19A	−179.9 (12)
C23—Fe1—C19—C20	117.0 (11)		

### Hydrogen-bond geometry (Å, °)

**Table:**

*D*—H···*A*	*D*—H	H···*A*	*D*···*A*	*D*—H···*A*
C10—H10B···O3	0.97	2.47	2.877 (3)	105
C9—H9···O1	0.93	2.58	3.128 (3)	118
C10A—H10C···O1^i^	0.98	2.56	3.371 (3)	140
N5—H5A···O3^ii^	0.86	1.99	2.840 (3)	168

Symmetry codes: (i) *x*, −*y*+1/2, *z*+1/2; (ii) −*x*+1, −*y*, −*z*.
